# Adjuvant cytokine-induced killer cell immunotherapy for hepatocellular carcinoma: a propensity score-matched analysis of real-world data

**DOI:** 10.1186/s12885-019-5740-z

**Published:** 2019-05-31

**Authors:** Jun Sik Yoon, Byeong Geun Song, Jeong-Hoon Lee, Hyo Young Lee, Sun Woong Kim, Young Chang, Yun Bin Lee, Eun Ju Cho, Su Jong Yu, Dong Hyun Sinn, Yoon Jun Kim, Joon Hyeok Lee, Jung-Hwan Yoon

**Affiliations:** 1 Department of Internal Medicine and Liver Research Institute, Seoul National University College of Medicine, Seoul, South Korea; 2 Department of Internal Medicine, Busan Paik Hospital, Inje University College of Medicine, Busan, South Korea; 3 Department of Medicine, Samsung Medical Center, Sungkyunkwan University School of Medicine, Seoul, South Korea; 4 Department of Internal Medicine, Eulji General Hospital, Eulji University School of Medicine, Seoul, South Korea; 5 Department of Internal Medicine, Digestive Disease Center, Institute for Digestive Research, Soonchunhyang University College of Medicine, Seoul, South Korea

**Keywords:** Hepatocelluar carcinoma, Cytokine-induced killer cell, Adjuvant immunotherapy, Recurrence-free survival, Overall survival

## Abstract

**Background:**

Several randomized controlled trials have shown that adjuvant immunotherapy with autologous cytokine-induced killer (CIK) cells prolongs recurrence-free survival (RFS) after curative treatment for hepatocellular carcinoma (HCC). We investigated the efficacy of adjuvant immunotherapy with activated CIK cells in real-world clinical practice.

**Methods:**

A total of 59 patients who had undergone curative surgical resection or radiofrequency ablation for stage I or II HCC, and subsequently received adjuvant CIK cell immunotherapy at two large-volume centers in Korea were retrospectively included. Propensity score matching with a 1:1 ratio was conducted to avoid possible bias, and 59 pairs of matched control subjects were also generated. The primary endpoint was RFS and the secondary endpoints were overall survival and safety.

**Results:**

The median follow-up duration was 28.0 months (interquartile range, 22.9–42.3 months). In a univariable analysis, the immunotherapy group showed significantly longer RFS than the control group (hazard ratio [HR], 0.42; 95% CI, 0.22–0.80; log-rank *P =* 0.006). The median RFS in the control group was 29.8 months, and the immunotherapy group did not reach a median RFS. A multivariable Cox proportional hazard analysis showed that immunotherapy was an independent predictor for HCC recurrence (adjusted HR, 0.38; 95% CI, 0.20–0.73; *P =* 0.004). The overall incidence of adverse events in the immunotherapy group was 16/59 (27.1%) and no patient experienced a grade 3 or 4 adverse event.

**Conclusions:**

The adjuvant immunotherapy with autologous CIK cells after curative treatment safely prolonged the RFS of HCC patients in a real-world setting.

**Electronic supplementary material:**

The online version of this article (10.1186/s12885-019-5740-z) contains supplementary material, which is available to authorized users.

## Background

Hepatocellular carcinoma (HCC) is an aggressive cancer that most often occurs in patients with chronic liver disease and cirrhosis. The use of active surveillance programs for these high-risk patients can detect HCC at an early stage and improve survival [1–3]. Surgical liver resection and radiofrequency ablation (RFA) are the most frequently used curative treatment options for early stage HCC patients with preserved liver function [4, 5]. However, approximately 70% of HCC patients who undergo surgical resection or RFA will experience recurrence within 5 years [6]. Together with hepatic decompensation, the recurrence of HCC are the major causes of mortality in these patients [7]. Therefore, the effective adjuvant therapies that can be applied after curative treatments to reduce recurrence are urgently needed.

Cytokine-induced killer (CIK) cells are a class of non-major histocompatibility complex (MHC)-restricted T lymphocytes that comprise CD3+/CD56+ cells, CD3+/CD56- cytotoxic T cells, and CD3+/CD56- natural killer (NK) cells [8]. They are generated ex vivo by incubation of human mononuclear cells with stimulative anti-CD3 antibody and interleukin (IL)-2 [9]. Because CIK cells can recognize MHC-lost or -downregulated malignant cells, which escape immune surveillance, rendered CIK cells can be a therapeutic option for tumors [10]. Several randomized controlled trials (RCTs) have shown that adjuvant CIK cell immunotherapy given after curative treatment for HCC reduces the rate of HCC recurrence with minimal side effects [11–14]. We previously reported an RCT that used a commercialized autologous CIK cell-based immunotherapeutic agent (ImmunCell-LC®; Green Cross Cell Corp, Seoul, Korea) manufactured in a Good Medical Practice (GMP)-certified facility. In that study, adjuvant CIK cell immunotherapy prolonged both recurrence-free survival (RFS) and overall survival (OS) [12]. Up until now, ImmunCell-LC® has been the only CIK cell agent commercially available.

This study was designed to assess whether the previously reported efficacy and safety of using ImmunCell-LC® as an adjuvant therapy after curative HCC treatment could be reproduced in a real-world clinical setting.

## Methods

### Patients

Patients who underwent a potentially curative treatment (surgical resection or RFA) for HCC were eligible for this study. The patients were assigned to two groups: those who had received adjuvant immunotherapy with a CIK cell agent (the immunotherapy group) and those who had not received any adjuvant treatment (the control group). HCC was diagnosed by histologic examination or radiologic imaging studies, according to guidelines of the American Association for the Study of Liver Diseases [15]. The study inclusion criteria were as follows: 1) stage I or II HCC based on radiologic findings described in the 6th edition of the American Joint Committee on Cancer (AJCC) Staging Manual; 2) Child-Pugh class A liver function; 3) an Eastern Cooperative Oncology Group performance status score ≤ 1. The study exclusion criteria were as follows: 1) Previous history of HCC treatment within 2 years prior to curative treatment; 2) less than 3 months of CIK infusion for patients in the immunotherapy group; 3) having received other adjuvant therapies for patients in the control group. Any patient who received CIK cell immunotherapy in a previous clinical trial was not included in this study.

### Study design and treatment protocol

The study was performed in accordance with the Declaration of Helsinki and approved by institutional review board of Seoul National University Hospital (SNUH, Seoul, Korea) and Samsung Medical Center (SMC, Seoul, Korea). This phase 4 clinical study was a multicenter, open-labeled trial conducted at two large-volume tertiary hospitals in Korea: SNUH and SMC. All enrolled patients underwent surgical resection or RFA as a curative treatment for HCC and had a medically-confirmed complete response. Patients in the immunotherapy group were enrolled from two centers (SNUH and SMC). As the two centers showed comparable treatment outcomes (RFS and OS) after curative treatment for HCC in our recent study (unpublished data), we enrolled patients in the control group from one center (SNUH).

For preparation of the individualized CIK cell agent, 150 mL of peripheral blood was collected from each patient in the immunotherapy group 2–3 weeks before initiating immunotherapy. Peripheral blood mononuclear cells (PBMCs) were extracted by leukapheresis and then expanded for 12–21 days with cytokines (IL-2 and anti-CD3 monoclonal antibody) according to our previously reported protocol [12]. A 200 mL aliquot of the prepared CIK cell agent was injected intravenously into patients in the immunotherapy group over a period of 60 min; after which, the patients were observed for at least 30 min to identify any immediate side effects. The CIK cell agent was scheduled to be injected 16 times over a period of 59 weeks (4 treatments every 1 week, 4 treatments every 2 weeks, 4 treatments every 4 weeks, and 4 treatments every 8 weeks). Treatment was discontinued when HCC recurrence was detected.

### Endpoints and treatment evaluation

The primary endpoint of this study was RFS, which was calculated from the date of curative treatment to the first HCC recurrence or death from any cause. The secondary endpoints were OS and safety. OS was calculated from the date of curative treatment to death from any cause. The date used for all-cause mortality was obtained from patient medical records and from the Korean Ministry of Government Administration and Home Affairs. The data cut-off date was March 31, 2019. To assess the safety of the CIK cell agent, AEs (adverse events) were investigated from the date of initiating adjuvant CIK cell immunotherapy until the end of the study or patient drop-out in the immunotherapy group. AEs were graded according to National Cancer Institute Common Terminology Criteria for Adverse Events, Version 3.0.

The baseline laboratory findings for patients in both groups were collected between 1 and 3 months after curative treatment, because acute liver function abnormalities may occur immediately after treatment. Laboratory results for patients in the immunotherapy group were collected prior to starting the adjuvant treatment with the CIK cell agents. Treatment evaluations were performed by dynamic computed tomography or magnetic resonance imaging every 3 months for the first 24 months and every 3–6 months thereafter in both groups.

### Statistical analysis

A 1:1 ratio propensity score (PS) matching analysis was performed to reduce selection bias due to differences in baseline characteristics between the two groups. A PS was calculated for each patient based on a multivariable logistic regression model. The variables in the model included age, sex, treatment modality, HCC stage, number of HCCs, size of the HCC, underlying liver disease, cirrhosis, prothrombin time, platelet count, lymphocyte-to-monocyte ratio, serum levels of alpha-fetoprotein (AFP), protein induced by vitamin K absence (PIVKA)-II, aspartate aminotransferase (AST), alanine aminotransferase (ALT), total bilirubin, and albumin. The nearest neighbor method was used in match selection. That method, matches a patients with another patient whose PS is closest to their own [16]. Standardized mean differences were calculated to ensure that the variables in the two groups were well-balanced.

Data are expressed as a median value (IQR) or n (%). Comparisons between patients in the two groups were assessed using Mann-Whitney’s *U*-test for continuous data and the chi-square or Fisher’s exact test for categorical data. Survival curves (RFS and OS) were calculated by the Kaplan-Meier method, and a log-rank test or a Firth’s method were used for group comparisons. Crude HRs were calculated using the Cox proportional hazard model. A Forest plot of subgroup analyses was constructed to compare the ongoing effect of immunotherapy on the RFS of patients in the immunotherapy group with those of patients in the control group. Using statistically significant variables in a univariable analysis, multivariable Cox proportional hazard analysis was performed to identify factors associated with RFS. *P*-values < 0.05 were considered statistically significant. R package MatchIt, Version 3.4.4 (R Foundation for Statistical Computing, Vienna, Austria) was used for the PS matching. All other statistical analyses were performed using IBM SPSS Statistics for Windows, Version 23.0 (IBM, Corp., Armonk, NY, USA).

## Results

### Patients

**Fig. 1:**
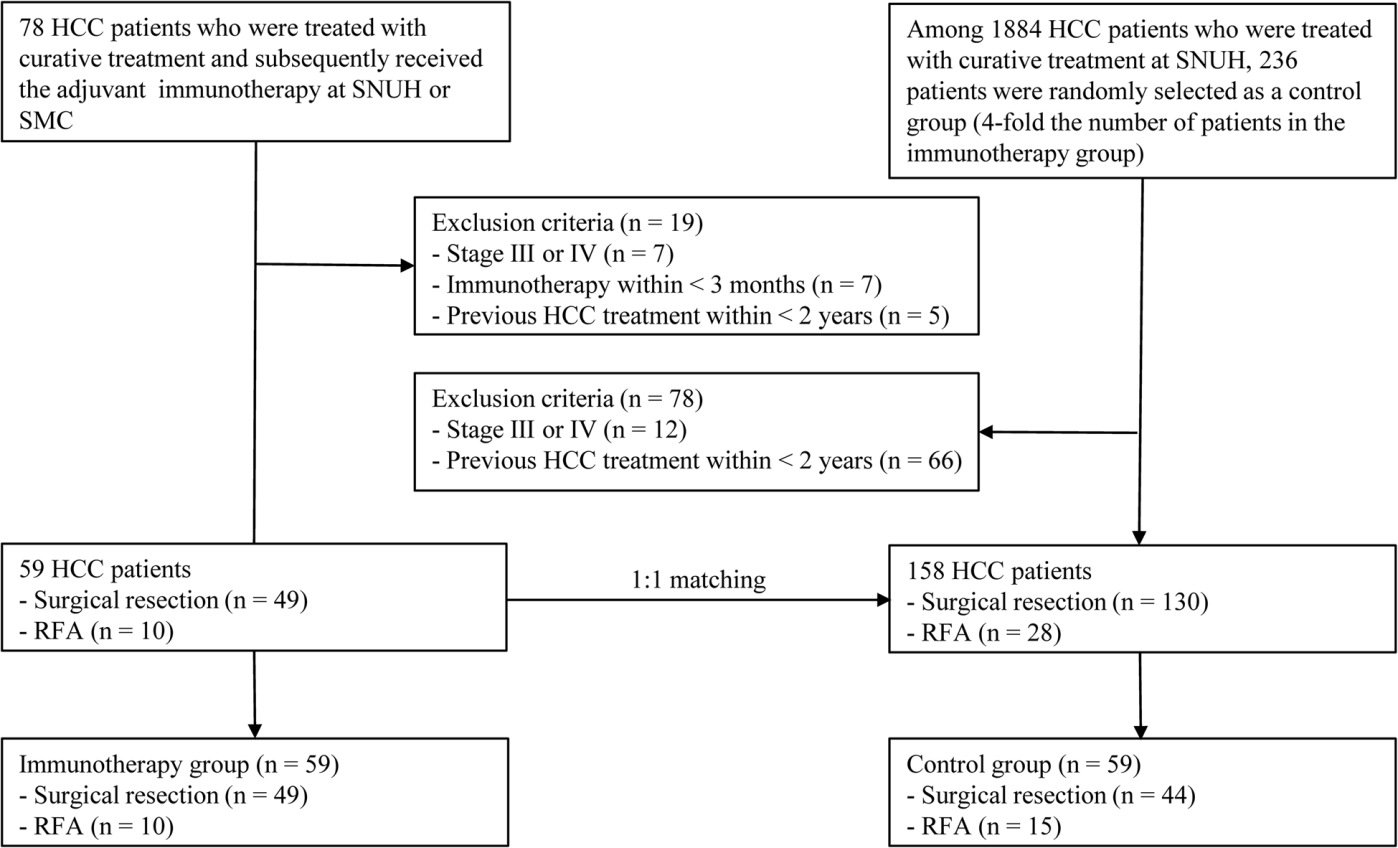
CONSORT Diagram. SNUH, Seoul National University Hospital; SMC, Samsung Medical Center; HCC, hepatocellular carcinoma; RFA, radiofrequency ablation

From February 2014 to December 2017, a total of 78 HCC patients underwent curative surgical resection or RFA and then received the CIK cell agents at two large-volume medical centers in Korea. A total of 59 patients satisfied the study inclusion criteria and were assigned to the immunotherapy group: 24 at SNUH and 35 at SMC. During the same period, a total of 1884 HCC patients underwent curative treatments with no adjuvant therapy at a single medical center (SNUH). We randomly selected 236 of those patients, which was 4-fold the number of patients in the immunotherapy group, by using a PS matching model consisting of two variables (age and sex). Among those selected patients, 158 satisfied the study inclusion criteria. We then performed a 1:1 PS matching for those 158 patients and 59 patients in the immunotherapy group. Finally, 59 pairs of patients were selected for inclusion in the immunotherapy group and control group (Fig. 1). Among 59 patients in the immunotherapy group, 5 had previous history of treatments with a median time from the prior treatment of 46 months (range, 24–55 months). Whereas, all patients in the control group did not have a previous history of treatment. The median follow-up durations for patients in the immunotherapy group and control group were 31.5 months (interquartile range [IQR], 23.1–47.0 months) and 26.9 months (IQR, 22.8–40.7 months), respectively.

There were several differences in baseline characteristics between the immunotherapy group and control group prior to the PS matching. The HCC stage (*P* = 0.018) and prevalence of cirrhosis (*P* < 0.001) were significantly higher in the immunotherapy group, and the serum levels of AST, albumin, and total bilirubin were significantly different in the two groups (all *P* < 0.05). After PS matching, all the variables of baseline characteristics were comparable between the two groups, with a ≤ 0.25 standardized mean difference (Table 1).

**Table 1:** Baseline characteristics of the immunotherapy group and control group before and after propensity score matching

	Before propensity-matched population			After propensity-matched population			
	Immunotherapy (*n* = 59)	Control group (*n* = 158)	*P* value	Immunotherapy (*n* = 59)	Control group (*n* = 59)	*P* value	*d* ^a^
Male sex, N (%)	45 (76.3%)	125 (79.1%)	0.79	45 (76.3%)	46 (78.0%)	1.00	0.040
Age, yrs	57.0 (48.5–62.0)	59.0 (52.0–65.0)	0.28	57.0 (48.5–62.0)	59.0 (52.0–65.0)	0.30	0.158
Treatment modality			1.00			0.37	0.224
RFA	10 (16.9%)	28 (17.7%)		10 (16.9%)	15 (25.4%)		
Surgical resection	49 (83.1%)	130 (82.3%)		49 (83.1%)	44 (74.6%)		
HCC stage, N (%)			0.02			0.46	0.170
Stage I	25 (42.4%)	97 (61.4%)		25 (42.4%)	30 (50.8%)		
Stage II	34 (57.6%)	61 (38.6%)		34 (57.6%)	29 (49.2%)		
Number of HCC, N (%)			0.64			1.00	< 0.001
< 3	57 (96.6%)	156 (98.7%)		57 (96.6%)	57 (96.6%)		
≥ 3	2 (3.4%)	2 (1.3%)		2 (3.4%)	2 (3.4%)		
Size of HCC, cm	2.9 (2.1–3.9)	2.5 (1.7–3.5)	0.07	2.9 (2.1–3.9)	2.3 (1.9–3.6)	0.23	0.115
Cause of liver disease, N (%)			0.76			1.00	< 0.001
HBV infection	53 (89.8%)	136 (86.1%)		53 (89.8%)	53 (89.8%)		
HCV infection	2 (3.4%)	8 (5.1%)		2 (3.4%)	2 (3.4%)		
Others	4 (6.8%)	14 (8.9%)		4 (6.8%)	4 (6.8%)		
Cirrhosis, N (%)	35 (59.3%)	48 (30.4%)	< 0.001	35 (59.3%)	31 (52.5%)	0.58	0.137
α-fetoprotein level, ng/mL	3.8 (2.6–6.2)	3.6 (2.5–7.1)	0.56	3.8 (2.6–6.2)	4.1 (2.7–8.5)	0.67	0.091
PIVKA-II, mAU/mL	20.0 (16.0–23.0)	19.0 (16.0–26.0)	0.52	20.0 (16.0–23.0)	20.0 (15.0–25.5)	0.58	0.102
Aspartate aminotransferase level, IU/L	31.0 (26.0–40.0)	26.0 (22.0–34.0)	0.005	31.0 (26.0–40.0)	29.0 (24.0–37.5)	0.37	0.161
Alanine aminotransferase level, IU/L	30.0 (17.5–38.0)	22.5 (17.0–33.0)	0.12	30.0 (17.5–38.0)	22.0 (18.5–33.0)	0.29	0.206
Alkaline phosphatase level, IU/L	78.0 (65.5–95.0)	85.0 (70.0–101.0)	0.16	78.0 (65.5–95.0)	87.0 (73.0–103.0)	0.06	–
Albumin level, g/dL	4.2 (4.0–4.5)	4.1 (3.8–4.3)	0.02	4.2 (4.0–4.5)	4.2 (3.8–4.5)	0.70	0.126
Total bilirubin level, mg/dL	0.6 (0.5–0.8)	0.7 (0.6–0.9)	0.01	0.6 (0.5–0.8)	0.7 (0.6–0.9)	0.06	0.245
Prothrombin time, INR	1.1 (1.0–1.1)	1.1 (1.0–1.2)	0.39	1.1 (1.0–1.1)	1.1 (1.0–1.2)	0.72	0.051
Creatinine level, mg/dL	0.9 (0.8–1.0)	0.8 (0.7–0.9)	0.12	0.9 (0.8–1.0)	0.8 (0.7–0.9)	0.08	–
Platelet, ×10^3^/mm^3^	165.0 (123.0–221.0)	184.5 (136.0–233.0)	0.17	165.0 (123.0–221.0)	158.0 (130.5–200.0)	0.72	0.069
Lymphocyte to monocyte ratio	4.0 (3.0–5.2)	4.4 (3.6–5.7)	0.09	4.4 (3.6–5.7)	4.2 (2.8–5.4)	0.33	0.005

Data are expressed as n (%), median (interquartile range)

*RFA* radiofrequency ablation, *HCC* hepatocellular carcinoma, *HBV* hepatitis B virus, *HCV* hepatitis C virus, *PIVKA-II* protein induced by vitamin K absence-II, *INR* international normalized ratio

^a^A standardized mean difference (*d*) of < 0.1 indicated very small differences; 0.1–0.3, small differences; 0.3–0.5, moderate differences; > 0.5, considerable differences

### Recurrence-free survival

**Fig. 2:**
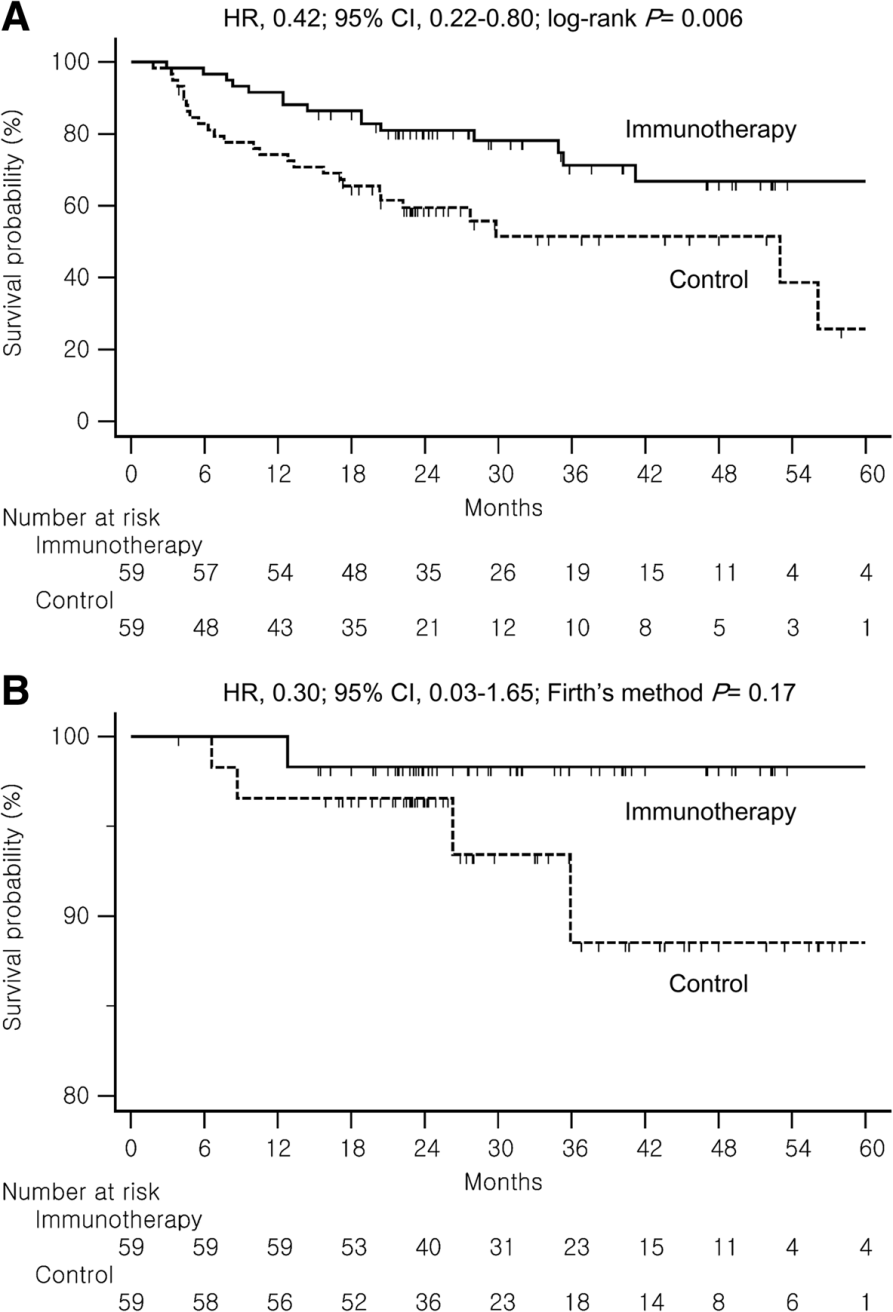
Kaplan-Meier estimates of recurrence-free survival (**a**) and overall survival (**b**). HR, hazard ratio; CI, confidence interval

**Fig. 3:**
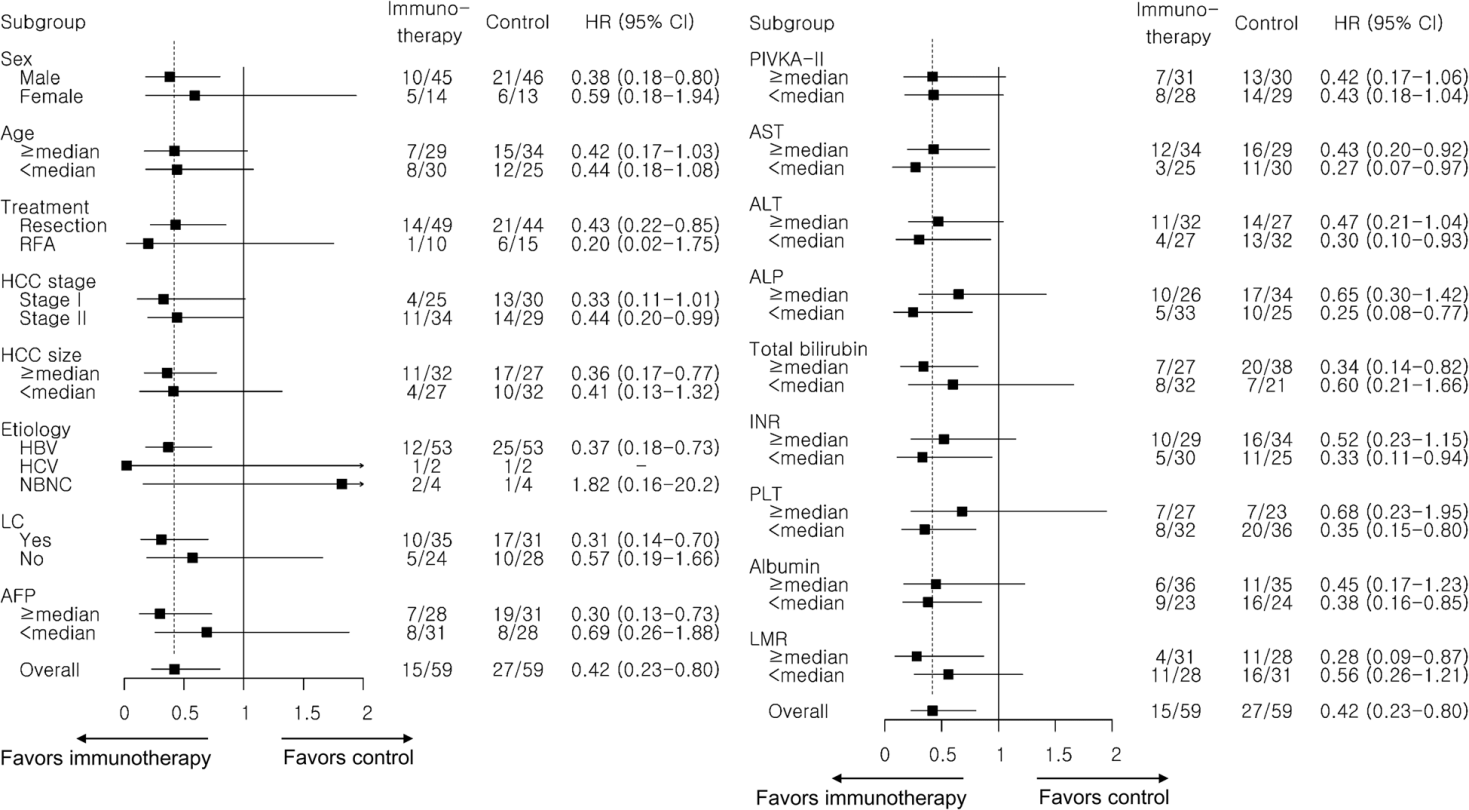
Recurrence-free survival in selected subsets. HR, hazard ratio; RFA, radiofrequency ablation; HCC, hepatocellular carcinoma; HBV, hepatitis B virus, HCV, hepatitis C virus, LC, liver cirrhosis; AFP, alpha-fetoprotein; PIVKA-II, protein induced by vitamin K absence-II; AST, aspartate aminotransferase; ALT, alanine aminotransferase; ALP, Alkaline phosphatase; INR, international normalized ratio; PLT, platelet; LMR, lymphocyte to monocyte ratio

In a univariable analysis, the immunotherapy group showed a significantly longer RFS than the control group (hazard ratio [HR], 0.42; 95% confidence interval [CI], 0.22–0.80; log-rank *P =* 0.006) (Fig. 2a). A median RFS was not reached in the immunotherapy group, and that in the control group was 29.8 months. There was a statistically significant difference in RFS between the two groups. Fifteen of the 59 patients (25.4%) in the immunotherapy group and 27 of the 59 patients (45.8%) in the control group experienced tumor recurrence or death during the study period. The crude HR for RFS in the immunotherapy group vs. the control group was 0.42 (95% CI, 0.22–0.80, *P =* 0.008). A Forest plot of the crude HRs with a 95% CI for RFS according to subgroup showed that the immunotherapy group had a longer RFS than the control group in all the subgroups, except etiology of HCC which was not statistically significant (Fig. 3).

In a univariable Cox proportional hazard analysis, RFS was significantly associated with immunotherapy, size of the HCC, lymphocyte to monocyte ratio, serum levels of AFP, AST, and albumin. (all *P* < 0.05). In a multivariable analysis with a stepwise selection, immunotherapy was an independent negative risk factor for tumor recurrence or death (adjusted HR, 0.38; 95% CI, 0.20–0.73, *P* = 0.004) along with the size of the HCC (Table 2). When we analyzed after excluding the 5 treatment-experienced patients, the adjusted HR of CIK cell immunotherapy was 0.38 (95% CI, 0.20–0.74; *P* = 0.005), which was comparable to that of the whole study population. We also performed additional analysis only in patients enrolled at one center (SNUH; 24 patients in the immunotherapy group vs. 59 patients in the control group) after excluding the 35 patients enrolled at SMC, and the adjusted HR (0.31; 95% CI, 0.11–0.87; *P* = 0.03) of the analysis was maintained in comparison with that of the whole study population.

**Table 2:** Factors associated with recurrence-free survival

	Univariable analysis		Multivariable analysis	
	HR (95% CI)	*P* value	HR (95% CI)	*P* value
Immunotherapy (Yes vs No)	0.42 (0.22–0.80)	0.008	0.38 (0.20–0.73)	0.004
Sex (male vs female)	0.93 (0.46–1.88)	0.85	–	–
Age (≥ 58 yrs vs < 58 yrs)^a^	0.94 (0.51–1.72)	0.84	–	–
Treatment modality (Resection vs RFA)	1.73 (0.76–3.95)	0.19	–	–
HCC stage (II vs I)	1.54 (0.83–2.87)	0.17	–	–
Number of HCC (≥ 3 vs < 3)	2.39 (0.57–9.95)	0.23	–	–
Size of HCC (≥ 2.75 cm vs < 2.75 cm)^a^	2.67 (1.39–5.11)	0.003	2.47 (1.25–4.90)	0.01
Cause of liver disease		0.51	–	–
HBV infection	1 (reference)			
HCV infection	0.78 (0.24–2.55)			
Others	1.77 (0.30–10.62)			
Cirrhosis (Yes vs No)	1.45 (0.77–2.73)	0.25	–	–
α-fetoprotein (≥ 4 ng/mL vs < 4 ng/mL)^a^	1.93 (1.03–3.61)	0.04	1.12 (0.54–2.31)	0.76
PIVKA-II (≥ 20 mAU/mL vs < 20 mAU/mL)^a^	0.74 (0.40–1.36)	0.34	–	–
Aspartate aminotransferase (≥ 30 IU/L vs < 30 IU/L)^a^	1.99 (1.04–3.79)	0.04	1.62 (0.81–3.24)	0.17
Alanine aminotransferase (≥ 27 IU/L vs < 27 IU/L)^a^	1.60 (0.86–2.97)	0.14	–	–
Alkaline phosphatase (≥ 83 IU/L vs < 83 IU/L)^a^	1.73 (0.92–3.27)	0.09	–	–
Albumin (≥ 4.2 g/dL vs < 4.2 g/dL)^a^	0.36 (0.19–0.68)	0.001	0.51 (0.25–1.04)	0.06
Total bilirubin (≥ 0.7 mg/dL vs < 0.7 mg/dL)^a^	1.40 (0.74–2.64)	0.31	–	–
Prothrombin time, INR (≥ 1.09 vs < 1.09)^a^	1.56 (0.83–2.90)	0.17	–	–
Creatinine (≥ 0.85 mg/dL vs < 0.85 mg/dL)^a^	0.82 (0.44–1.50)	0.52	–	–
Platelet (≥ 173 mm^3^ vs < 173 mm^3^)^a^	0.69 (0.36–1.32)	0.27	–	–
Lymphocyte to monocyte ratio (≥ 4.36 vs < 4.36)^a^	0.47 (0.25–0.89)	0.02	0.87 (0.40–1.90)	0.72

^a^Continuous variables are divided according to their median values

*RFA* radiofrequency ablation, *HCC* hepatocellular carcinoma, *HBV* hepatitis B virus, *HCV* hepatitis C virus, *PIVKA-II* protein induced by vitamin K absence-II, *INR* international normalized ratio, *HR* hazard ratio

Among 42 patients (15 in the immunotherapy group and 27 in the control group) with tumor recurrence, patients received additional treatment with various modalities including transarterial chemoembolization, RFA, surgical resection, liver transplantation, sorafenib and external radiation therapy (Additional file 1).

### Overall survival

Until the data cut-off date, 5 death had occurred in the entire study population; 1 patient died in the immunotherapy group and 4 patient died in the control group. The 1 patient in the immunotherapy group died of recurrent HCC. The patients in the control group died of recurrent HCC (3 patients) or new primary lung cancer (1 patient). There was no statistically significant difference in OS between the two groups (*P* = 0.17 as determined by Firth’s method) (Fig. 2b).

### Safety

AEs occurred in 16 of the 59 (27.1%) patients in the immunotherapy group, and all the AEs were of grade 1 or 2 in severity. Fatigue (6.8%) was the most frequently reported AE, followed by pyrexia (5.1%) (Table 3). All the AEs were self-limiting and improved with conservative management. No patient delayed or stopped their immunotherapy due to an AE. No infectious complications or allergic reactions were observed in patients in the immunotherapy group.

**Table 3:** Adverse events in the immunotherapy group

Adverse event	Immunotherapy (*n* = 59)	
	Grade 1 or 2	Grade 3 or 4
Overall incidence	16 (27.1%)	0
Anorexia	1 (1.7%)	0
Nausea	1 (1.7%)	0
Vomiting	2 (3.4%)	0
Pruritis	2 (3.4%)	0
Chills	2 (3.4%)	0
Fatigue	4 (6.8%)	0
Pyrexia	3 (5.1%)	0
Productive cough	1 (1.7%)	0

## Discussion

The question addressed by the present study was whether the previously reported efficacy and safety of administering a CIK cell agent as adjuvant therapy after curative HCC treatment could be reproduced in real-world clinical practice. The main finding of the study was that the adjuvant CIK cell agent prolonged RFS with minimal side effects in HCC patients who had undergone curative treatment. Although the efficacy of the adjuvant CIK cell agent has already been demonstrated in the previous RCT [12], the data in real-world setting has not been evaluated yet. The participants in RCT, who are enrolled in a clear set of inclusion and exclusion criteria may not represent real-world population, which may lead to a bias [17]. Therefore, it is important to validate the positive results of CIK cell immunotherapy in real-world clinical practice. Moreover, CIK cell immunotherapy is not incorporated into the recent clinical guidelines due to lack of real-world evidence [18, 19]. Our study provides real-world evidence that the efficacy of adjuvant CIK cell immunotherapy in RCT was maintained in real-world clinical practice.

CIK cells are non-MHC-restricted T lymphocytes that, can be expanded ex vivo from a patients’ PBMCs, following stimulation with anti-CD3 antibody and IL-2, and can exhibit anti-tumor effects in vivo [8–10]. CIK cells can display not only a T lymphocyte-like phenotype but also an NK cell-like phenotype [20]. Thus, CIK cells can exert the natural cytotoxic function of NK cells, and recognize tumor cells even in the absence of surface antigens [21]. Tumor cells can escape immune surveillance in various ways, and a loss of antigenicity is one key mechanism of escape [22]. MHC is the major tissue-antigen that allows the immune system to recognize tumor cells, and the loss of MHC causes immune escape. However, CIK cells can recognize tumor cells and kill them without a prior exposure or priming without MHC restriction. Due to these unique anti-tumor effects, CIK cells have been widely studied as a treatment for various cancers, including HCC [23, 24].

Immunotherapy provided with CIK cells is reported to be more effective when applied a low tumor burden stage and in an adjuvant setting [25]. Therefore, we planned to administer the adjuvant CIK cell immunotherapy only to patients with early stage HCC. In this study, the schedule for CIK cell administration and the use of the commercialized CIK cell agent were the same as those described in the RCT that we previously reported [12]. Several previous RCTs [11–14] and retrospective studies [26, 27] consistently reported that adjuvant CIK cell immunotherapy prolongs RFS and/or OS when administered after curative treatment for HCC. However, the methods used to select the HCC patients, the schedule for CIK cell administration, and the methods used to prepare CIK cells differed in each study. Some studies enrolled HCC patients with more advanced stage and failed to show a beneficial effect of CIK cell immunotherapy on OS [11, 13, 14]. The optimal schedule for CIK cell administration has also been controversial; however, it was reported that the maximum beneficial effect on OS was achieved when more than 8 cycles of CIK cell administration were performed [27]. In most of the previous studies, the investigators produced the CIK cells using their own cultivation techniques. In contrast, we used commercialized CIK cell agents that had been manufactured in a GMP-certified facility that had standard operating procedures and strict quality control standards. We believe that administering more than 8 cycles of the commercialized CIK cell agent to early stage HCC patients in an adjuvant setting may improve the efficacy of CIK cell immunotherapy.

The adjusted HR for the RFS produced by CIK cell immunotherapy in our study was 0.38, which was lower than that of previously reported studies (HR of 0.59–0.67) [11–13]. Previous RCTs showed that CIK cell immunotherapy was more effective at reducing the rate of early recurrence (within the first 24 months) than late recurrence (beyond 24 months) [11–14]. Moreover, the CIK cell immunotherapy was more effective when administered to patients with a low tumor burden [25]. The present study had a relatively short follow-up duration (median = 28.0 months) and included only stage I and stage II patients. Therefore, the CIK cell immunotherapy used in this study was more effective at prolonging RFS than the CIK cell immunotherapies used in previous studies. However, we did not show that the CIK immunotherapy prolonged the OS of the HCC patients. Because this study had a short follow-up duration, there was only 1 patient death in the immunotherapy group and 4 patients death in the control group. A longer follow-up duration is needed to demonstrate any survival benefit of the CIK cell immunotherapy.

The overall incidence of AEs in the immunotherapy group was 27.1% and there was no grade 3 or 4 AEs. In previous studies, the reported overall incidence of AEs varied from 3.5 to 62%, and the majority were light fever at grade 1 or 2 in severity, which was consistent with this study [11–14, 26, 27]. Because CIK cells are manufactured by the ex vivo culture of autologous PMBCs stimulated with cytokines, they are less toxic and produce no graft-versus-host effect [28–30]. No patients in this study had to stop or delay their CIK cell immunotherapy due to an AE. These results imply that CIK cell immunotherapy is safe and well tolerated therapeutic modality.

There are several limitations to our study. First, probably because of the small number of enrolled patients and short follow-up duration, we failed to show any survival benefit of the CIK cell immunotherapy for HCC patients. However, we showed that the adjuvant CIK cell immunotherapy was a potent therapeutic modality for reducing recurrence, which is the most frequent cause of death among HCC patients. Second, this study had a retrospective design. Therefore, there might have been bias when selecting patients for the control group. However, in order to reduce selection bias, we used the PS matching technique to select the control group patients. After PS matching, there were no significant differences between the baseline characteristics of patients in the immunotherapy group and control group. Although there was no significant difference, the median tumor size of the immunotherapy group was larger than the control group. Considering that tumor size is one of the most important factor of HCC recurrence after curative treatment [31–33], it might be notable that the HR for the RFS produced by CIK cell immunotherapy in our study was lower than that of the RCT that we previously reported [12]. This result may refute the criticism that the therapeutic effect of CIK cell immunotherapy was over-estimated because patients in the immunotherapy group had significantly smaller tumors than patients in the control group in our previous RCT [34]. Third, we did not identify predictors for which patients would be responders in the immunotherapy group. We believe that CIK cell immunotherapy should be considered as an adjuvant treatment for patients with early stage tumors. However, even if adjuvant CIK cell immunotherapy were performed on these selected patients, 15 out of 59 patients (25.4%) of the patients would experience recurrence. Further studies which compare pretreatment and post-treatment factors in the responders and non-responders are needed.

## Conclusions

In conclusion, we showed that adjuvant CIK cell immunotherapy prolonged the RFS of HCC patients who had undergone curative treatment in a real-world clinical setting. All the AEs associated with the immunotherapy were mild to moderate in severity and self-limiting.

## Additional file

Additional file 1: **TableS1.** The most frequent post-recurrent treatment modality was transarterial chemoembolization, followed by radiofrequency ablation and surgical resection. (DOCX 15 kb)

## Data Availability

The datasets used and/or analyzed during the current study are available from the corresponding author upon reasonable request.

## Abbreviations

AEs: Adverse events
AFP: Alpha-fetoprotein
AJCC: American Joint Committee on Cancer
ALT: Alanine aminotransferase
AST: Aspartate aminotransferase
CIK: Cytokine-induced killer
GMP: Good Medical Practice
HCC: Hepatocellular carcinoma
IL: Interleukin
MHC: Major histocompatibility complex
NK: Natural killer
OS: Overall survival
PBMCs: Peripheral blood mononuclear cells
PIVKA-II: Protein induced by vitamin K absence-II
PS: Propensity score
RCTs: Randomized controlled trials
RFA: Radiofrequency ablation
RFS: Recurrence-free survival
