# Supplementary material for: Adjuvant cytokine-induced killer cell immunotherapy for hepatocellular carcinoma: a propensity score-matched analysis of real-world data
Source: BMC Cancer. 2019 May 31;19:523. doi: 10.1186/s12885-019-5740-z (PMC6543598; doi:10.1186/s12885-019-5740-z)
Supplement: Supplementary file 1 — TableS1. The most frequent post-recurrent treatment modality was transarterial chemoembolization, followed by radiofrequency ablation and surgical resection. (DOCX 15 kb) [file 12885_2019_5740_MOESM1_ESM.docx]

Supplementary Table. Post-recurrent treatment modalities in the immunotherapy group and the control group.

| Treatment modalities | Immunotherapy (n=15) | Control (n=27) |
| --- | --- | --- |
| Transarterial chemoembolization | 14 | 25 |
| Radiofrequency ablation | 8 | 12 |
| Surgical resection | 3 | 5 |
| Liver transplantation | 0 | 1 |
| Sorafenib | 2 | 2 |
| Radiation therapy | 0 | 1 |
| Total | 27 | 46 |
